# Public health impact and cost-effectiveness of dairy products supplemented with vitamin D in prevention of osteoporotic fractures

**DOI:** 10.1186/s13690-015-0099-3

**Published:** 2015-12-14

**Authors:** Olivier Ethgen, Mickaël Hiligsmann, Nansa Burlet, Jean-Yves Reginster

**Affiliations:** Department of Public Health, Epidemiology and Health Economics, University of Liege, Liege, Belgium; Department of Health Services Research, Maastricht University, Maastricht, The Netherlands; Department of Public Health, Epidemiology and Health Economics, University of Liege, Liege, Belgium; Department of Public Health, Epidemiology and Health Economics, University of Liege, Liege, Belgium

**Keywords:** Osteoporosis, Cost-effectiveness, Dairy products, Vitamin D, Fracture, Osteoporosis

## Abstract

**Background:**

Dietary sources of calcium and vitamin D are recommended as a first-line strategy in prevention of osteoporosis-related fractures but their public health and economic impact has never been studied.

**Methods:**

We designed a population-based model to forecast the potential health outcomes and medical effectiveness of the daily administration of dairy supplements containing 800 IU of vitamin D and 1 g of calcium in cohorts of subjects, from both genders, aged 50, 60, 70 and 80 years. Annual costs of dairy products were tested at €150, €250 and €350.

**Results:**

In total, the daily intake of vitamin-D rich dairy products reduces by 30,376 and 16,105 events the number of osteoporotic fractures in women and men respectively and permits to gain 6605 and 6144 life-years, in women and men respectively. This intervention is cost-effective from 70 years on in the general population and from 60 years on in patients at increased risk of osteoporotic fractures.

**Conclusion:**

The recommendation to use dairy products as the preferred source of calcium and vitamin D in aging males and females is supported by public health and health economic analyses.

**Electronic supplementary material:**

The online version of this article (doi:10.1186/s13690-015-0099-3) contains supplementary material, which is available to authorized users.

## Background

Osteoporosis (OP) is defined as a systemic disease characterized by low bone mass and microarchitectural deterioration of bone tissue, with a consequent increase in bone fragility and susceptibility to fractures (Fx) [1, 2]. Collectively, all OP Fx account for 2.7 million Fx in men and women in Europe [2]. In 2010, the direct costs of OP Fx in the five largest EU countries was evaluated at €29 billion and at €38.7 billion in the 27 countries [3, 4]. All OP Fx but forearm are associated with increased mortality [5, 6]. In 2012, the number of deaths causally related to OP Fx in the European union, was estimated at 43,000 [4], of which 50 % were due to hip Fx, 28 % to clinical vertebral and 22 % to other Fx [2]. This high societal and personal burden poses challenges to public health and physicians [2]. There is a high prevalence of vitamin D insufficiency in the elderly and dietary calcium is low in many postmenopausal OP women [7–10], notwithstanding most data suggest that dietary intakes of key bone nutrients such as calcium, vitamin D and proteins contribute to bone health and reduce the risk of Fx later in life [2, 11]. Dietary sources of nutrients are the preferred option and pharmacological supplementation should only be targeted to those individuals who do not get sufficient calcium from their diet and who are at increased risk for OP [2]. Recent European consensus recommend adequate vitamin D intake of 800 IU/day as well as calcium intake of 1000 mg/day [7, 12]. Until now, few studies targeted the cost-effectiveness of calcium and vitamin D supplementation in OP [13, 14] and even less [15] assessed the public health and economic impact of fortified dairy products given to the general elderly population.

## Methods

We designed a population-based model to forecast and compare the potential population health outcomes and medical costs following an appropriate daily intake of calcium/vitamin D in Belgium. Appropriate daily intake was obtained by the administration, on top of the regular diet, of a daily dairy supplementation containing 1000 mg of calcium and 800 IU of vitamin D [7, 12]. This amount can be achieved with 1 l of milk (35 g of proteins), 100 g of Comte cheese (28 g proteins) or 4 yogurts (20 g of proteins). The model was actually an extension of an extensively published and validated cost-effectiveness microsimulation model of OP management [13, 16–18]. The model was reengineered to accommodate multiple age cohorts, constituent of a population [19].

We used the median variant population projection 2014–2060 from the SPF Economie, the official source for socio-demographic data in Belgium [20, 21]. Projections for 4 female and 4 male cohorts aged 50, 60, 70 and 80 years on the 1st of January 2015 were extracted (birth cohorts 1965, 1955, 1945 and 1935).

The 8 cohorts were stratified according to their baseline risk of OP Fx: general population, population with osteoporosis (as defined by a BMD T-score ≤ 2.5) and population with a prevalent vertebral or hip Fx. Baseline prevalence estimates per age were derived from literature [3]. Prevalence assumptions and resulting numbers of individuals with OP and a prevalent hip or vertebral Fx in age strata are presented in Tables 1 and 2.

**Table 1:** Prevalence of osteoporosis and vertebral/hip fracture in 4 birth cohorts of women

Age	Total^a^	General		Osteoporosis^b^		Prevalent fracture^c^	
		%	N	%	N	%	N
50	83,691	93.3 %	78,084	6.6 %	5524	0.1 %	84
60	70,979	81.9 %	58,132	16.3 %	11,570	1.8 %	1278
70	52,754	67.0 %	35,345	28.6 %	15,08	84.4 %	2321
80	41,157	40.1 %	16,504	47.2 %	19,426	12.7 %	5,2271

^a^SPF Economie. Ref. 20–21

^b^BMD T-Score ≤ 2.5. Ref. 22

^c^Prevalent hip or vertebral Fx. Ref 22

**Table 2:** Prevalence of osteoporosis and vertebral/hip fracture in 4 birth cohorts of men

Age	Total^a^	General		Osteoporosis^b^		Prevalent fracture^c^	
		%	N	%	N	%	N
50	85,138	97.2 %	82,754	2.6 %	2214	0.2 %	170
60	69,040	91.9 %	63,448	6.7 %	4626	1.4 %	967
70	47,455	88.5 %	41,998	8.5 %	4034	3.0 %	1424
80	29,235	57.0 %	16,664	36.0 %	10,525	7.0 %	2046

^a^SPF Economie. Ref. 20–21

^b^BMD T-Score ≤ 2.5. Ref. 22

^c^Prevalent hip or vertebral Fx. Ref 22

In the model, the occurrence of Fx at individual ages is modelled through a health-state transition process (Markov model). Individuals begin either in the general population within the non-Fx state, in the OP population within the non-Fx state or in the OP population within the prevalent Fx state. Individuals then progress according to their baseline characteristics and age-specific Fx risk through the non-Fx stage, hip Fx stage, vertebral Fx stage, wrist Fx stage or other Fx stage.

As it is a population-based model, the size of each cohort corresponds to the actual size of the corresponding age cohort in the Belgian population. Hence, the adapted model differs from the previous microsimulation model on the target population dynamics as it does not assume a hypothetical and microsimulated cohort of patients but projects the impact of calcium/vitamin D supplementation, through dairy products, on a specific age cohort over its respective remaining lifetime. All transition probabilities were recalculated as annual transition probabilities as the original microsimulation model uses 6-month probabilities.

Each year, all individuals can die from age-specific background mortality from any health state.

As for the population size estimates above, the age-specific background mortality rates were taken from the SPF Economie [20, 21]. We assumed that hip Fx increased death probabilities by 5.5, as evidenced in a meta-analysis [22]. The same excess mortality was assumed for vertebral Fx but not for wrist and others Fx.

The risk of hip Fx was derived from the Belgian national database of hospital bills [23]. Since the incidence of non-hip Fx was not known, we applied the age-specific ratio of index Fx to hip Fx in Belgium as found in Sweden [24]. All estimates were interpolated using exponential regression to obtain age-specific risk of Fx. We assumed an increased risk of Fx for individuals who had a prior Fx at the same location. These increased relative risks were 2.3, 4.4, 3.3 and 1.9 for hip, vertebral, wrist and others Fx, respectively [16, 25, 26].

Calcium/vitamin D supplementation effectiveness in the reduction of Fx incidence was taken from a recent study [13] that reviewed and synthesized meta-analyses previously published on the subject. Calcium/vitamin D supplementation has been shown to reduce the risk of hip Fx by 18 % (*RR* = 0.82 95 % CI:[7.1–0.94] in a meta-analysis of 6 trials including 45.509 patients) [27], the risk of vertebral Fx by 13 % (*RR* = 0.87 95 % Cl:[0.75–1.01] in a meta-analysis including 45.164 patients) [28] and the risk of non-hip non-vertebral Fx by 20 % (*RR* = 0.80 95 % Cl:[0.72–0.89] in a meta-analysis of 9 trials including 32.285 patients) [29]. Patients were assumed to follow calcium/vitamin D supplementation for their remaining lifetime. The protective effect of calcium/vitamin D supplementation on Fx risk was thus assumed to remain constant over time.

The perspective for the cost calculation is that of the Belgian health care payers (government and patients) as recommended in the Belgian pharmacoeconomics guideline [30]. Only direct medical costs were taken into account. The hospitalization cost of hip Fx was retrieved from the Belgian national database of hospital bills for the year 2007 [13, 17, 18]. Extra costs in the year following a hip Fx were derived from a prospective study of 159 women [31]. The costs of non-hip Fx were estimated relative to hip Fx [32]. We assumed that non-hip Fx were not associated with long-term costs. As for the risks of Fx, all cost estimates were interpolated using power or polynomial regression to obtain age-specific cost of Fx. In the absence of official pricing data, as opposed to pharmaceutical for instance, the price of the supplementation with dairy products was derived from the observed market price of vitamin D-enriched milk and yogurt and was assumed at €250 per year and tested between €150 and €350. All costs were expressed in €2014 using the national health price index [20, 21] and were discounted at 3 % per annum.

The model has a lifetime horizon for each age cohort considered.

We projected the number of Fx avoided and life-years gained (LYG) for each age cohort, from 50 to 80 years of age in 2015 (born between 1935 and 1965). We also computed the cost-per-Fx avoided and the cost-per-LYG for each age cohort.

## Results

Tables 3 and 4 summarize the projected health and cost impacts of the recommended dairy daily intake versus the absence of appropriate intake for women and men aged 50, 60, 70 and 80 years in 2015 in Belgium. In total, 30,376 Fx would be avoided and 6605 LY would be gained in the 4 age cohorts of women over their remaining lifetime. In men, the number of Fx avoided and LYG amounted 16,105 and 6144, respectively. The greatest absolute gains were seen in the general population segment, regardless of gender of baseline age.

**Table 3:** Health and cost impact of recommended CA/Vit D dietary intake in women

Fracture avoided	LYG							
	50y	60y	70y	80y	50y	60y	70y	80y
General	9928	6739	3497	1219	1305	1242	1040	706
Osteoporosis1	1051	1967	2131	1944	86	240	472	991
Prevalent fracture2	29	406	594	871	2	38	97	386
Cost per fracture avoided	Cost per LYG							
	50y	60y	70y	80y	50y	60y	70y	80y
General								
Ca/Vit.D €150/year	€ 22,462	€ 19,094	€ 14,467	€ 8064	€ 170,818	€ 103,630	€ 48,663	€ 13,924
Ca/Vit.D €250/year	€ 38,993	€ 33,990	€ 27,182	€ 17,815	€ 296,532	€ 184,479	€ 91,430	€ 30,759
Ca/Vit.D €350/year	€ 55,524	€ 48,887	€ 39,896	€ 27,566	€ 422,245	€ 265,329	€ 134,197	€ 47,594
Osteoporosis^a^								
Ca/Vit.D €150/year	€ 13,617	€ 11,144	€ 7690	€ 3057	€ 165,846	€ 91,326	€ 34,719	€ 5997
Ca/Vit.D €250/year	€ 24,655	€ 21,277	€ 16,547	€ 10,149	€ 300,277	€ 174,359	€ 74,707	€ 19,910
Ca/Vit.D €350/year	€ 35,692	€ 31,409	€ 25,404	€ 17,240	€ 434,709	€ 257,392	€ 114,696	€ 33,822
Prevalent fracture^b^								
Ca/Vit.D €150/year	€ 5941	€ 3711	€ 1049	€ 0	€ 83,603	€ 39,497	€ 6392	€ 0
Ca/Vit.D €250/year	€ 11,988	€ 9090	€ 5856	€ 1492	€ 168,701	€ 96,744	€ 35,687	€ 3369
Ca/Vit.D €350/year	€ 18,035	€ 14,469	€ 10,663	€ 5532	€ 253,799	€ 153,992	€ 64,982	€ 12,493

^a^BMD T-score ≤ 2.5

^b^Hip or vertebral

**Table 4:** Health and cost impact of recommended CA/Vit D dietary intake in men

	Fracture avoided				LYG			
	50y	60y	70y	80y	50y	60y	70y	80y
General	6383	4315	2351	675	1727	1540	1216	571
Osteoporosis^a^	279	498	342	601	49	131	149	479
Prevalent fracture^b^	40	195	223	203	6	41	80	155
	Cost per fracture avoided				Cost per LYG			
	50y	60y	70y	80y	50y	60y	70y	80y
General								
Ca/Vit.D €150/year	€ 35,684	€ 31,117	€ 24,794	€ 16,389	€ 131,903	€ 87,190	€ 47,950	€ 19,377
Ca/Vit.D €250/year	€ 61,073	€ 54,030	€ 44,275	€ 31,334	€ 225,753	€ 151,392	€ 85,627	€ 37,048
Ca/Vit.D €350/year	€ 86,463	€ 76,943	€ 63,757	€ 46,279	€ 319,603	€ 215,595	€ 123,304	€ 54,719
Osteoporosis^a^								
Ca/Vit.D €150/year	€ 20,250	€ 17,654	€ 13,935	€ 8873	€ 115,310	€ 66,932	€ 32,000	€ 11,124
Ca/Vit.D €250/year	€ 35,749	€ 32,068	€ 26,716	€ 19,327	€ 203,563	€ 121,582	€ 61,349	€ 24,231
Ca/Vit.D €350/year	€ 51,248	€ 46,482	€ 39,497	€ 29,781	€ 291,816	€ 176,231	€ 90,697	€ 37,337
Prevalent fracture^b^								
Ca/Vit.D €150/year	€ 9214	€ 7204	€ 4546	€ 1148	€ 62,625	€ 34,065	€ 12,649	€ 1505
Ca/Vit.D €250/year	€ 17,483	€ 14,815	€ 11,293	€ 6799	€ 118,823	€ 70,057	€ 31,423	€ 8916
Ca/Vit.D €350/year	€ 25,751	€ 22,427	€ 18,040	€ 12,451	€ 175,022	€ 106,049	€ 50,197	€ 16,327

^a^BMD T-score ≤ 2.5

^b^Hip or vertebral

The cost-per-Fx avoided was below €60,000 in women (maximum of €55,524 in the general population aged 50 years with a cost of supplementation at €350 per year) and below €90,000 in men (maximum of €86,463 in the general population aged 50 years with a cost of supplementation at €350 per year). In both gender, the cost-per-Fx avoided declined markedly with age and baseline risk of Fx.

The cost-per-LYG (cost-effectiveness) was above €100,000 in the 50 years age cohort for both women and men, except in the prevalent Fx risk-group. The cost-per-LYG declined along aging cohorts and in the older cohort (80 years), the cost-per-LYG generally fell below €50,000. In all instances, the cost-per-LYG tended to be more favorable (i.e. lower) in older and riskier cohorts.

Detailed results for each specific age cohort of women and men are presented as Additional file 1.

## Discussion

Our study suggest that by ensuring an adequate repletion in calcium and vitamin D to the general population, by the administration of dairy products providing 800 IU of vitamin D and 1000 mg of calcium daily, it is possible to reduce the number of OP-related FX and to provide a substantial number of LYG, in people for both genders, currently aged from 50 to 80 years old. Our results also suggest that this intervention is cost/effective in both genders, above the age of 70 years in the general population and from 60 years and above in subjects at increased risk of OP. Although, the clinical efficacy of pharmacological or non-pharmacological interventions may be consistent across settings, their impact on public health or their economic value may differ, owing to differences in epidemiologic variations, interventions-related costs and effectiveness of available alternative strategies [33, 34]. In Europe, OP accounts for more disability and LY lost than all other major musculoskeletal conditions, except osteoarthritis but including rheumatoid arthritis [2]. With regards to neoplastic diseases, the burden of OP is greater than all sites of cancer with the exception of lung cancer [35]. Several studies assessed the efficacy and efficiency of pharmacological interventions in OP [36]. However, nutritional interventions whereas considered as first-line measures in most guidelines dedicated to the management of OP [1, 2, 7, 12] are less frequently studied in terms of their impact on public health or health resources utilization [15]. This study is the first to apply a validated cost-effectiveness microsimulation model [16], adapted to a public health perspective [19] to the outcomes of calcium and vitamin D administration, as dairy products, on reduction of Fx or on gain in LY and to determine whether such an intervention may be considered cost-effective in the general population and in individuals at increased risk of OP-related Fx. The scarcity of available resources and the increasing demand for healthcare require more rational assignment of resources and better definition of priorities. In this scenario, economic evaluations provide valuable information and LYG is widely recognized as an appropriate outcome [37]. Our results show that vitamin D-enriched dairy products, given to the general population, from the age of 50 years can spare more than 30,000 OP Fx in women and more than 15,000 OP Fx in men and save more than 6600 LY in women and more than 6100 LY in men. As expected, the cost-effectiveness of dairy product administration in OP, as it is the case for any intervention, is strongly influenced by the cost of the intervention and by the risk profile of the subject. No standard for upper threshold of cost per LYG, that would constitute an absolute value of the critical cost-effectiveness ratio, is explicitly fixed [38]. However, tentative guidelines place it in the range of $20,000 to $100,000 per LYG [39], while other authors consider that up to $166,000 per LYG, cost-efficiency is debatable [40].

The World Health Organization (WHO) suggests that interventions with a cost-effectiveness ratio between 1 and 3-fold de Gross Domestic Product (GDP) per capita of the country where the study is conducted are cost/effective [41]. In Belgium, GDP per capita accounted €37,857.75 in 2014. These figures, based on the WHO recommendations [41] suggest that cost-effective ratios below €120,000/LYG are acceptable.

The cost-effectiveness ratio of our intervention remains below or around €100,000/LYG in the general population at 70 years and above, while these values are observed from 60 years on, in subjects at increased risk of OP Fx except in the sensitivity analysis setting up the price of the dairy intervention at €350/year.

Like any health economic study, our work is based on a number of assumptions. The cost-effectiveness is critically dependent on the evidence for efficacy of the intervention. It could be argued that prospective, randomized controlled studies establishing the anti Fx efficacy of dairy product are currently missing [42]. Some cohort studies also generated unconclusive evidence on reduction of Fx following milk intake. However, these cohorts did not use any vitamin D-enriched dairy products [43–46], while our analysis is based on the intake of dairy products providing the amount of calcium and vitamin D, which are considered by most experts and recommendations from scientific organizations as appropriate to improve bone health [1, 2, 7, 12]. Divergent opinion, challenging the interest of calcium and vitamin D in OP [47] or reporting putative increase in adverse effects following calcium supplementation [48] appear to be insufficiently substantiated [49] and, at any rate more directed towards calcium pharmacological supplementation than against dairy products intake [48].

Our extrapolation of the anti-Fx efficacy obtained with pharmacological supplementation of calcium and vitamin D to dairy products providing the same amount of nutrients is indeed likely to be highly conservative. Several studies have documented the link between low intake of dairy foods and decreased bone mineral density or increased Fx risk [50, 51].

At every stage of life, adequate dietary intake of key bone nutrients including not only calcium and vitamin D but also proteins contributes to muscle and bone health, thereby reducing the risk of falls, OP and Fx later in life [12]. Dairy products are rich in proteins [52]. There is no clear-cut evidence that individuals aged more than 50 years old have a deficit in protein intake similar to what is reported for calcium and vitamin D [53]. However, it is possible that our analysis, based only on the beneficial effects of calcium and vitamin D on bone, without modelling the potential benefits of proteins underestimates the positive outcomes of dairy products intake.

Furthermore, it is well known that the efficacy of anti OP medications, including pharmacological supplements of calcium and vitamin D is decreased as a result of poor adherence [54]. The impact of low compliance and persistence on the cost-effectiveness of these treatments is well documented [55]. Dairy food has been shown to be an appropriate vehicle to provide calcium, with good compliance compared to pharmacological supplements [15, 56], which might have a positive effect on the effectiveness of our approach. This might also be emphasized because the putative deleterious effect of pharmacological calcium supplementation on myocardial infarction was never reported following dairy products intake. Eventually, different studies showed that calcium and vitamin D supplementation have other health benefits effects. So, studies suggested that calcium and vitamin D may reduce the risk for breast cancer mainly in premenopausal women [57] and colon cancer in older women [58]. A meta-analysis has also indicated that vitamin D may have a small beneficial effect on cardiovascular risk and mortality [59].

All these potential benefits might increase the number of LYG by our dairy product supplementation and, subsequently, improve the cost/effectiveness of the intervention by reducing the cost/LYG. Eventually, we only assessed beneficial outcomes generated by the calcium/vitamin D supplementation in terms of LYG. Improvements in quality of life and subsequent quality-adjusted LY were also reported following the Fx risk reduction induced by a dietary supplementation in calcium/vitamin D and reflect a qualitative benefit that is superimposed to the quantitative outcome assessed by the LYG.

## Conclusion

Dietary sources of nutrients are recommended as appropriate to ensure adequate calcium and vitamin D repletion. This recommendation, widely claimed to be justified in terms of efficacy is also supported by our public health impact and health economics analyses.

## Additional file

Additional file 1:
**Results in women and men.** (PDF 212 kb)

## Abbreviations

BMD: Bone mineral density
Fx: Facture
GDP: Gross domestic product
LYG: Life-years gained
LY: Life-years
OP: Osteoporosis
WHO: World Health Organization
