# Supplementary material for: Public health impact and cost-effectiveness of dairy products supplemented with vitamin D in prevention of osteoporotic fractures
Source: Arch Public Health. 2015 Dec 14;73:48. doi: 10.1186/s13690-015-0099-3 (PMC4677432; doi:10.1186/s13690-015-0099-3)
Supplement: Additional file 1: — Results in women and men. (PDF 212 kb) [file 13690_2015_99_MOESM1_ESM.pdf]

Supplementary file:

Detailed results obtained in terms of public health impact, life-years gain, cost and efficiency in men and women, from the general population, with densitometric osteoporosis and prevalent fracture, for a dairy supplementation costing €150/year, €250/year or €350/year, at the age of 50 years, 60 years, 70 years and 80 years.

Results in women

**Women**	*General*			*Osteoporosis*[1]			*Prevalent fracture*[2]		
50 years N=	78,084			5,524			84		
	**Without** recommended Ca/Vit.D intake	**With** recommended Ca/Vit.D intake	Incremental analysis	**Without** recommended Ca/Vit.D intake	**With** recommended Ca/Vit.D intake	Incremental analysis	**Without** recommended Ca/Vit.D intake	**With** recommended Ca/Vit.D intake	Incremental analysis
**Public health impact**									
**Number of new fractures**									
Hip	13,379	11,637	-1,742	1,801	1,563	-239	53	46	-7
Vertebral	11,937	10,731	-1,206	1,446	1,307	-139	145	129	-16
Wrist	15,483	12,334	-3,149	1,503	1,201	-302	16	13	-3
Other	20,840	17,009	-3,831	2,085	1,714	-371	20	17	-3
Total	*61,638*	*51,710*	*-9,928*	*6,835*	*5,784*	*-1,051*	*233*	*205*	*-29*
**LYG**									
Without fracture	2,217,622	2,310,662	93,040	129,096	138,075	8,978	1,465	1,594	129
With ≥ 1 fracture	646,315	554,581	-91,734	73,262	64,370	-8,892	1,587	1,459	-127
Total	2,863,937	2,865,243	1,305	202,358	202,444	86	3,051	3,053	2
**Cost impact**									
**Fracture cost**	€ 157,121,822	€ 133,947,675	-€ 23,174,147	€ 21,343,639	€ 18,255,606	-€ 3,088,033	€ 672,220	€ 581,653	-€ 90,567
**Ca/Vit D supplementation**									
Ca/Vit.D €150/year	—	€ 246,173,613	€ 246,173,613	—	€ 17,392,804	€ 17,392,804	—	€ 262,476	€ 262,476
Ca/Vit.D €250/year	—	€ 410,289,354	€ 410,289,354	—	€ 28,988,007	€ 28,988,007	—	€ 437,461	€ 437,461
Ca/Vit.D €350/year	—	€ 574,405,096	€ 574,405,096	—	€ 40,583,210	€ 40,583,210	—	€ 612,445	€ 612,445
**Total**									
Ca/Vit.D €150/year	€ 157,121,822	€ 380,121,288	€ 222,999,465	€ 21,343,639	€ 35,648,410	€ 14,304,772	€ 672,220	€ 844,129	€ 171,910
Ca/Vit.D €250/year	€ 157,121,822	€ 544,237,029	€ 387,115,207	€ 21,343,639	€ 47,243,613	€ 25,899,974	€ 672,220	€ 1,019,114	€ 346,894
Ca/Vit.D €350/year	€ 157,121,822	€ 708,352,771	€ 551,230,949	€ 21,343,639	€ 58,838,816	€ 37,495,177	€ 672,220	€ 1,194,098	€ 521,878
**Efficiency**									
**Cost per fracture avoided**									
Ca/Vit.D €150/year			€ 22,462			€ 13,617			€ 5,941
Ca/Vit.D €250/year			€ 38,993			€ 24,655			€ 11,988
Ca/Vit.D €350/year			€ 55,524			€ 35,692			€ 18,035
**Cost per LYG**									
Ca/Vit.D €150/year			€ 170,818			€ 165,846			€ 83,603
Ca/Vit.D €250/year			€ 296,532			€ 300,277			€ 168,701
Ca/Vit.D €350/year			€ 422,245			€ 434,709			€ 253,799

[1] BMD T-score ≤ 2.5

[2] Hip or vertebral

**Women**		*General*			*Osteoporosis* [1]			*Prevalent fracture* [2]		
60 years	N=	58,132			11,570			1,278		
		**Without** recommended Ca/Vit.D intake	**With** recommended Ca/Vit.D intake	Incremental analysis	**Without** recommended Ca/Vit.D intake	**With** recommended Ca/Vit.D intake	Incremental analysis	**Without** recommended Ca/Vit.D intake	**With** recommended Ca/Vit.D intake	Incremental analysis
**Public health impact**										
**Number of new fractures**										
Hip		9,865	8,510	-1,355	3,699	3,179	-520	823	704	-119
Vertebral		8,122	7,288	-834	2,637	2,381	-256	1,901	1,694	-207
Wrist		9,535	7,641	-1,893	2,469	1,991	-478	192	158	-34
Other		14,353	11,696	-2,656	3,965	3,253	-712	282	237	-45
Total		*41,874*	*35,135*	*-6,739*	*12,770*	*10,803*	*-1,967*	*3,199*	*2,793*	*-406*
**LYG**										
Without fracture		1,193,512	1,248,201	54,690	197,309	211,574	14,266	15,914	17,422	1,509
With ≥ 1 fracture		379,043	325,595	-53,448	114,881	100,855	-14,026	18,290	16,819	-1,471
Total		1,572,555	1,573,797	1,242	312,189	312,429	240	34,203	34,241	38
**Cost impact**										
**Fracture cost**		€ 145,876,574	€ 123,969,213	-€ 21,907,361	€ 53,719,906	€ 45,745,195	-€ 7,974,711	€ 12,579,057	€ 10,808,711	-€ 1,770,346
**Ca/Vit D supplementation**										
Ca/Vit.D €150/year		—	€ 150,577,844	€ 150,577,844	—	€ 29,896,068	€ 29,896,068	—	€ 3,278,112	€ 3,278,112
Ca/Vit.D €250/year		—	€ 250,963,074	€ 250,963,074	—	€ 49,826,780	€ 49,826,780	—	€ 5,463,520	€ 5,463,520
Ca/Vit.D €350/year		—	€ 351,348,304	€ 351,348,304	—	€ 69,757,492	€ 69,757,492	—	€ 7,648,928	€ 7,648,928
**Total**										
Ca/Vit.D €150/year		€ 145,876,574	€ 274,547,058	€ 128,670,483	€ 53,719,906	€ 75,641,263	€ 21,921,357	€ 12,579,057	€ 14,086,823	€ 1,507,766
Ca/Vit.D €250/year		€ 145,876,574	€ 374,932,287	€ 229,055,713	€ 53,719,906	€ 95,571,975	€ 41,852,069	€ 12,579,057	€ 16,272,231	€ 3,693,175
Ca/Vit.D €350/year		€ 145,876,574	€ 475,317,517	€ 329,440,942	€ 53,719,906	€ 115,502,687	€ 61,782,781	€ 12,579,057	€ 18,457,639	€ 5,878,583
**Efficiency**										
**Cost per fracture avoided**										
Ca/Vit.D €150/year				€ 19,094			€ 11,144			€ 3,711
Ca/Vit.D €250/year				€ 33,990			€ 21,277			€ 9,090
Ca/Vit.D €350/year				€ 48,887			€ 31,409			€ 14,469
**Cost per LYG**										
Ca/Vit.D €150/year				€ 103,630			€ 91,326			€ 39,497
Ca/Vit.D €250/year				€ 184,479			€ 174,359			€ 96,744
Ca/Vit.D €350/year				€ 265,329			€ 257,392			€ 153,992

[1] BMD T-score ≤ 2.5

[2] Hip or vertebral

**Women**		General			Osteoporosis [1]			Prevalent fracture [2]		
70 years	N=	35,345			15,088			2,321		
		**Without** recommended Ca/Vit.D intake	**With** recommended Ca/Vit.D intake	Incremental analysis	**Without** recommended Ca/Vit.D intake	**With** recommended Ca/Vit.D intake	Incremental analysis	**Without** recommended Ca/Vit.D intake	**With** recommended Ca/Vit.D intake	Incremental analysis
**Public health impact**										
**Number of new fractures**										
	Hip	5,786	4,937	-849	4,464	3,797	-668	1,405	1,192	-212
	Vertebral	4,153	3,720	-433	2,718	2,451	-266	2,533	2,262	-271
	Wrist	4,300	3,473	-827	2,271	1,850	-422	245	204	-41
	Other	7,478	6,090	-1,388	4,302	3,527	-775	436	367	-69
	Total	*21,716*	*18,220*	*-3,497*	*13,755*	*11,624*	*-2,131*	*4,620*	*4,026*	*-594*
**LYG**										
	Without fracture	473,844	496,761	22,916	171,366	183,720	12,354	18,939	20,801	1,862
	With ≥ 1 fracture	155,747	133,870	-21,877	95,779	83,897	-11,882	21,430	19,666	-1,764
	Total	629,591	630,630	1,040	267,145	267,617	472	40,369	40,467	97
**Cost impact**										
**Fracture cost**		€ 104,348,122	€ 88,248,543	-€ 16,099,579	€ 77,827,567	€ 65,904,556	-€ 11,923,011	€ 25,187,971	€ 21,527,520	-€ 3,660,452
**Ca/Vit D supplementation**										
	Ca/Vit.D €150/year	—	€ 66,687,482	€ 66,687,482	—	€ 28,308,848	€ 28,308,848	—	€ 4,283,503	€ 4,283,503
	Ca/Vit.D €250/year	—	€ 111,145,803	€ 111,145,803	—	€ 47,181,414	€ 47,181,414	—	€ 7,139,171	€ 7,139,171
	Ca/Vit.D €350/year	—	€ 155,604,124	€ 155,604,124	—	€ 66,053,979	€ 66,053,979	—	€ 9,994,839	€ 9,994,839
**Total**										
	Ca/Vit.D €150/year	€ 104,348,122	€ 154,936,025	€ 50,587,903	€ 77,827,567	€ 94,213,405	€ 16,385,838	€ 25,187,971	€ 25,811,022	€ 623,051
	Ca/Vit.D €250/year	€ 104,348,122	€ 199,394,346	€ 95,046,224	€ 77,827,567	€ 113,085,970	€ 35,258,403	€ 25,187,971	€ 28,666,691	€ 3,478,719
	Ca/Vit.D €350/year	€ 104,348,122	€ 243,852,667	€ 139,504,545	€ 77,827,567	€ 131,958,536	€ 54,130,969	€ 25,187,971	€ 31,522,359	€ 6,334,388
**Efficiency**										
**Cost per fracture avoided**										
	Ca/Vit.D €150/year			€ 14,467			€ 7,690			€ 1,049
	Ca/Vit.D €250/year			€ 27,182			€ 16,547			€ 5,856
	Ca/Vit.D €350/year			€ 39,896			€ 25,404			€ 10,663
**Cost per LYG**										
	Ca/Vit.D €150/year			€ 48,663			€ 34,719			€ 6,392
	Ca/Vit.D €250/year			€ 91,430			€ 74,707			€ 35,687
	Ca/Vit.D €350/year			€ 134,197			€ 114,696			€ 64,982

[1] BMD T-score ≤ 2.5

[2] Hip or vertebral

**Women**	*General*			*Osteoporosis* [1]			*Prevalent fracture* [2]		
80 years N=	16,504			19,426			5,227		
	**Without** recommended Ca/Vit.D intake	**With** recommended Ca/Vit.D intake	Incremental analysis	**Without** recommended Ca/Vit.D intake	**With** recommended Ca/Vit.D intake	Incremental analysis	**Without** recommended Ca/Vit.D intake	**With** recommended Ca/Vit.D intake	Incremental analysis
**Public health impact**									
**Number of new fractures**									
Hip	2,434	2,057	-377	4,664	3,937	-727	2,508	2,124	-384
Vertebral	1,387	1,243	-144	2,302	2,078	-225	3,257	2,928	-329
Wrist	1,226	1,000	-226	1,700	1,399	-301	320	270	-50
Other	2,566	2,094	-472	3,880	3,187	-692	701	593	-109
Total	*7,613*	*6,394*	*-1,219*	*12,546*	*10,602*	*-1,944*	*6,786*	*5,916*	*-871*
**LYG**									
Without fracture	120,816	126,708	5,892	124,397	132,775	8,378	24,015	26,288	2,273
With ≥ 1 fracture	37,536	32,350	-5,186	59,140	51,753	-7,387	22,777	20,889	-1,888
Total	158,351	159,058	706	183,537	184,528	991	46,792	47,178	386
**Cost impact**									
**Fracture cost**	€ 50,623,134	€ 42,621,636	-€ 8,001,499	€ 94,073,800	€ 79,333,364	-€ 14,740,436	€ 51,056,413	€ 43,560,110	-€ 7,496,303
**Ca/Vit D supplementation**									
Ca/Vit.D €150/year	—	€ 17,835,406	€ 17,835,406	—	€ 20,684,515	€ 20,684,515	—	€ 5,277,277	€ 5,277,277
Ca/Vit.D €250/year	—	€ 29,725,677	€ 29,725,677	—	€ 34,474,192	€ 34,474,192	—	€ 8,795,461	€ 8,795,461
Ca/Vit.D €350/year	—	€ 41,615,947	€ 41,615,947	—	€ 48,263,868	€ 48,263,868	—	€ 12,313,646	€ 12,313,646
**Total**									
Ca/Vit.D €150/year	€ 50,623,134	€ 60,457,042	€ 9,833,907	€ 94,073,800	€ 100,017,879	€ 5,944,079	€ 51,056,413	€ 48,837,387	-€ 2,219,026
Ca/Vit.D €250/year	€ 50,623,134	€ 72,347,312	€ 21,724,178	€ 94,073,800	€ 113,807,556	€ 19,733,756	€ 51,056,413	€ 52,355,571	€ 1,299,158
Ca/Vit.D €350/year	€ 50,623,134	€ 84,237,583	€ 33,614,449	€ 94,073,800	€ 127,597,233	€ 33,523,433	€ 51,056,413	€ 55,873,756	€ 4,817,343
**Efficiency**									
**Cost per fracture avoided**									
Ca/Vit.D €150/year			€ 8,064			€ 3,057			-€ 2,548
Ca/Vit.D €250/year			€ 17,815			€ 10,149			€ 1,492
Ca/Vit.D €350/year			€ 27,566			€ 17,240			€ 5,532
**Cost per LYG**									
Ca/Vit.D €150/year			€ 13,924			€ 5,997			-€ 5,755
Ca/Vit.D €250/year			€ 30,759			€ 19,910			€ 3,369
Ca/Vit.D €350/year			€ 47,594			€ 33,822			€ 12,493

[1] BMD T-score ≤ 2.5

[2] Hip or vertebral

Results in men

**Men**		General			Osteoporosis [1]			Prevalent fracture [2]		
50 years	N=	82,754			2,214			170		
		**Without** recommended Ca/Vit.D intake	**With** recommended Ca/Vit.D intake	Incremental analysis	**Without** recommended Ca/Vit.D intake	**With** recommended Ca/Vit.D intake	Incremental analysis	**Without** recommended Ca/Vit.D intake	**With** recommended Ca/Vit.D intake	Incremental analysis
**Public health impact**										
**Number of new fractures**										
	Hip	6,953	5,925	-1,028	390	332	-58	61	52	-9
	Vertebral	8,194	7,282	-912	376	337	-39	183	162	-22
	Wrist	2,832	2,324	-509	99	82	-16	6	5	-1
	Other	19,532	15,598	-3,934	832	665	-166	47	38	-8
	Total	*37,511*	*31,128*	*-6,383*	*1,695*	*1,416*	*-279*	*296*	*256*	*-40*
**LYG**										
	Without fracture	2,340,324	2,405,376	65,052	54,049	56,787	2,737	3,310	3,543	233
	With ≥ 1 fracture	406,240	342,916	-63,325	19,210	16,521	-2,688	2,274	2,047	-227
	Total	2,746,565	2,748,291	1,727	73,259	73,308	49	5,585	5,591	6
**Cost impact**										
**Fracture cost**		€ 94,785,681	€ 79,466,425	-€ 15,319,256	€ 5,290,541	€ 4,453,853	-€ 836,688	€ 865,599	€ 738,095	-€ 127,505
**Ca/Vit D supplementation**										
	Ca/Vit.D €150/year	—	€ 243,099,646	€ 243,099,646	—	€ 6,489,084	€ 6,489,084	—	€ 495,950	€ 495,950
	Ca/Vit.D €250/year	—	€ 405,166,076	€ 405,166,076	—	€ 10,815,139	€ 10,815,139	—	€ 826,583	€ 826,583
	Ca/Vit.D €350/year	—	€ 567,232,507	€ 567,232,507	—	€ 15,141,195	€ 15,141,195	—	€ 1,157,216	€ 1,157,216
**Total**										
	Ca/Vit.D €150/year	€ 94,785,681	€ 322,566,071	€ 227,780,390	€ 5,290,541	€ 10,942,937	€ 5,652,396	€ 865,599	€ 1,234,044	€ 368,445
	Ca/Vit.D €250/year	€ 94,785,681	€ 484,632,502	€ 389,846,820	€ 5,290,541	€ 15,268,992	€ 9,978,452	€ 865,599	€ 1,564,677	€ 699,078
	Ca/Vit.D €350/year	€ 94,785,681	€ 646,698,932	€ 551,913,251	€ 5,290,541	€ 19,595,048	€ 14,304,507	€ 865,599	€ 1,895,310	€ 1,029,711
**Efficiency**										
**Cost per fracture avoided**										
	Ca/Vit.D €150/year			€ 35,684			€ 20,250			€ 9,214
	Ca/Vit.D €250/year			€ 61,073			€ 35,749			€ 17,483
	Ca/Vit.D €350/year			€ 86,463			€ 51,248			€ 25,751
**Cost per LYG**										
	Ca/Vit.D €150/year			€ 131,903			€ 115,310			€ 62,625
	Ca/Vit.D €250/year			€ 225,753			€ 203,563			€ 118,823
	Ca/Vit.D €350/year			€ 319,603			€ 291,816			€ 175,022

[1] BMD T-score ≤ 2.5

[2] Hip or vertebral

**Men**	General			Osteoporosis [1]			Prevalent fracture [2]		
60 years N=	63,448			4,626			967		
	**Without** recommended Ca/Vit.D intake	**With** recommended Ca/Vit.D intake	Incremental analysis	**Without** recommended Ca/Vit.D intake	**With** recommended Ca/Vit.D intake	Incremental analysis	**Without** recommended Ca/Vit.D intake	**With** recommended Ca/Vit.D intake	Incremental analysis
**Public health impact**									
**Number of new fractures**									
Hip	5,028	4,261	-767	746	632	-114	318	269	-49
Vertebral	5,634	4,997	-638	680	608	-72	866	763	-103
Wrist	1,829	1,501	-328	169	141	-28	26	22	-4
Other	12,905	10,322	-2,582	1,432	1,148	-284	219	180	-39
Total	*25,396*	*21,082*	*-4,315*	*3,027*	*2,529*	*-498*	*1,429*	*1,234*	*-195*
**LYG**									
Without fracture	1,273,490	1,311,283	37,793	81,154	85,256	4,103	13,284	14,255	971
With ≥ 1 fracture	233,672	197,419	-36,253	28,204	24,233	-3,971	9,220	8,290	-930
Total	1,507,162	1,508,702	1,540	109,358	109,489	131	22,503	22,544	41
**Cost impact**									
**Fracture cost**	€ 86,181,399	€ 72,147,634	-€ 14,033,764	€ 12,357,772	€ 10,381,261	-€ 1,976,511	€ 5,494,117	€ 4,673,605	-€ 820,512
**Ca/Vit D supplementation**									
Ca/Vit.D €150/year	—	€ 148,291,460	€ 148,291,460	—	€ 10,770,887	€ 10,770,887	—	€ 2,223,469	€ 2,223,469
Ca/Vit.D €250/year	—	€ 247,152,434	€ 247,152,434	—	€ 17,951,478	€ 17,951,478	—	€ 3,705,781	€ 3,705,781
Ca/Vit.D €350/year	—	€ 346,013,408	€ 346,013,408	—	€ 25,132,069	€ 25,132,069	—	€ 5,188,094	€ 5,188,094
**Total**									
Ca/Vit.D €150/year	€ 86,181,399	€ 220,439,095	€ 134,257,696	€ 12,357,772	€ 21,152,148	€ 8,794,376	€ 5,494,117	€ 6,897,074	€ 1,402,957
Ca/Vit.D €250/year	€ 86,181,399	€ 319,300,069	€ 233,118,670	€ 12,357,772	€ 28,332,739	€ 15,974,967	€ 5,494,117	€ 8,379,386	€ 2,885,269
Ca/Vit.D €350/year	€ 86,181,399	€ 418,161,042	€ 331,979,644	€ 12,357,772	€ 35,513,330	€ 23,155,558	€ 5,494,117	€ 9,861,699	€ 4,367,582
**Efficiency**									
**Cost per fracture avoided**									
Ca/Vit.D €150/year			€ 31,117			€ 17,654			€ 7,204
Ca/Vit.D €250/year			€ 54,030			€ 32,068			€ 14,815
Ca/Vit.D €350/year			€ 76,943			€ 46,482			€ 22,427
**Cost per LYG**									
Ca/Vit.D €150/year			€ 87,190			€ 66,932			€ 34,065
Ca/Vit.D €250/year			€ 151,392			€ 121,582			€ 70,057
Ca/Vit.D €350/year			€ 215,595			€ 176,231			€ 106,049

[1] BMD T-score ≤ 2.5

[2] Hip or vertebral

**Men**		General			Osteoporosis [1]			Prevalent fracture [2]		
70 years	N=	41,998			4,034			1,424		
		**Without** recommended Ca/Vit.D intake	**With** recommended Ca/Vit.D intake	Incremental analysis	**Without** recommended Ca/Vit.D intake	**With** recommended Ca/Vit.D intake	Incremental analysis	**Without** recommended Ca/Vit.D intake	**With** recommended Ca/Vit.D intake	Incremental analysis
**Public health impact**										
**Number of new fractures**										
Hip		3,079	2,593	-487	562	473	-89	403	339	-64
Vertebral		3,135	2,775	-360	473	421	-52	946	833	-112
Wrist		930	763	-167	110	92	-19	29	25	-4
Other		6,758	5,420	-1,337	935	753	-182	244	201	-43
Total		*13,902*	*11,551*	*-2,351*	*2,080*	*1,739*	*-342*	*1,621*	*1,399*	*-223*
**LYG**										
Without fracture		538,319	554,727	16,407	46,088	48,304	2,216	12,626	13,541	915
With ≥ 1 fracture		98,586	83,394	-15,192	14,565	12,497	-2,067	8,154	7,319	-835
Total		636,905	638,121	1,216	60,653	60,801	149	20,780	20,860	80
**Cost impact**										
**Fracture cost**		€ 63,350,428	€ 52,937,255	-€ 10,413,173	€ 11,040,252	€ 9,251,049	-€ 1,789,203	€ 8,201,880	€ 6,960,596	-€ 1,241,283
**Ca/Vit D supplementation**										
Ca/Vit.D €150/year		—	€ 68,701,024	€ 68,701,024	—	€ 6,551,312	€ 6,551,312	—	€ 2,253,398	€ 2,253,398
Ca/Vit.D €250/year		—	€ 114,501,706	€ 114,501,706	—	€ 10,918,853	€ 10,918,853	—	€ 3,755,663	€ 3,755,663
Ca/Vit.D €350/year		—	€ 160,302,389	€ 160,302,389	—	€ 15,286,394	€ 15,286,394	—	€ 5,257,928	€ 5,257,928
**Total**										
Ca/Vit.D €150/year		€ 63,350,428	€ 121,638,279	€ 58,287,851	€ 11,040,252	€ 15,802,361	€ 4,762,109	€ 8,201,880	€ 9,213,994	€ 1,012,114
Ca/Vit.D €250/year		€ 63,350,428	€ 167,438,961	€ 104,088,533	€ 11,040,252	€ 20,169,902	€ 9,129,650	€ 8,201,880	€ 10,716,259	€ 2,514,380
Ca/Vit.D €350/year		€ 63,350,428	€ 213,239,644	€ 149,889,216	€ 11,040,252	€ 24,537,444	€ 13,497,191	€ 8,201,880	€ 12,218,524	€ 4,016,645
**Efficiency**										
**Cost per fracture avoided**										
Ca/Vit.D €150/year				€ 24,794			€ 13,935			€ 4,546
Ca/Vit.D €250/year				€ 44,275			€ 26,716			€ 11,293
Ca/Vit.D €350/year				€ 63,757			€ 39,497			€ 18,040
**Cost per LYG**										
Ca/Vit.D €150/year				€ 47,950			€ 32,000			€ 12,649
Ca/Vit.D €250/year				€ 85,627			€ 61,349			€ 31,423
Ca/Vit.D €350/year				€ 123,304			€ 90,697			€ 50,197

[1] BMD T-score ≤ 2.5

[2] Hip or vertebral

Men	General			Osteoporosis[1]			Prevalent fracture[2]		
80 years N=	16,664			10,525			2,046		
	**Without** recommended Ca/Vit.D intake	**With** recommended Ca/Vit.D intake	Incremental analysis	**Without** recommended Ca/Vit.D intake	**With** recommended Ca/Vit.D intake	Incremental analysis	**Without** recommended Ca/Vit.D intake	**With** recommended Ca/Vit.D intake	Incremental analysis
**Public health impact**									
**Number of new fractures**									
Hip	1,065	892	-173	1,127	945	-182	432	364	-68
Vertebral	922	815	-106	843	750	-93	818	725	-93
Wrist	241	197	-43	182	151	-31	27	23	-4
Other	1,821	1,468	-353	1,546	1,251	-294	222	185	-38
Total	*4,048*	*3,373*	*-675*	*3,697*	*3,096*	*-601*	*1,498*	*1,296*	*-203*
**LYG**									
Without fracture	115,544	119,014	3,470	66,744	69,624	2,880	10,066	10,743	677
With ≥ 1 fracture	19,183	16,284	-2,899	17,004	14,604	-2,400	5,175	4,653	-522
Total	134,727	135,298	571	83,748	84,227	479	15,241	15,395	155
**Cost impact**									
**Fracture cost**	€ 24,650,853	€ 20,578,458	-€ 4,072,395	€ 25,095,539	€ 21,004,331	-€ 4,091,208	€ 9,873,841	€ 8,387,996	-€ 1,485,845
**Ca/Vit D supplementation**									
Ca/Vit.D €150/year	—	€ 15,142,382	€ 15,142,382	—	€ 9,423,416	€ 9,423,416	—	€ 1,718,486	€ 1,718,486
Ca/Vit.D €250/year	—	€ 25,237,304	€ 25,237,304	—	€ 15,705,693	€ 15,705,693	—	€ 2,864,143	€ 2,864,143
Ca/Vit.D €350/year	—	€ 35,332,225	€ 35,332,225	—	€ 21,987,970	€ 21,987,970	—	€ 4,009,800	€ 4,009,800
**Total**									
Ca/Vit.D €150/year	€ 24,650,853	€ 35,720,840	€ 11,069,987	€ 25,095,539	€ 30,427,747	€ 5,332,208	€ 9,873,841	€ 10,106,482	€ 232,641
Ca/Vit.D €250/year	€ 24,650,853	€ 45,815,762	€ 21,164,908	€ 25,095,539	€ 36,710,024	€ 11,614,485	€ 9,873,841	€ 11,252,139	€ 1,378,298
Ca/Vit.D €350/year	€ 24,650,853	€ 55,910,683	€ 31,259,830	€ 25,095,539	€ 42,992,301	€ 17,896,762	€ 9,873,841	€ 12,397,796	€ 2,523,955
**Efficiency**									
**Cost per fracture avoided**									
Ca/Vit.D €150/year			€ 16,389			€ 8,873			€ 1,148
Ca/Vit.D €250/year			€ 31,334			€ 19,327			€ 6,799
Ca/Vit.D €350/year			€ 46,279			€ 29,781			€ 12,451
**Cost per LYG**									
Ca/Vit.D €150/year			€ 19,377			€ 11,124			€ 1,505
Ca/Vit.D €250/year			€ 37,048			€ 24,231			€ 8,916
Ca/Vit.D €350/year			€ 54,719			€ 37,337			€ 16,327

[1] BMD T-score ≤ 2.5

[2] Hip or vertebral
